# The Significance of Pericardial Fluid in Cases of Presumed Overlying

**Published:** 1910-10-01

**Authors:** J. H. Heaney


### The General Practitioner's Column.

[Contributions to this Column are invited, and, if accepted, will be paid for.]

### THE SIGNIFICANCE OF PERICARDIAL FLUID IN CASES OF PRESUMED OVERLYING.

By J. H. HEANEY, M.B. Irel.

A short time ago it was stated on good authority that in cases of presumed overlying a careful post-mortem search often reveals certain conditions which are sufficient in themselves to account for death, and which were then considered the primary cause. The frequent presence of pericardial fluid was emphasised. As far as I know—and I have purposely avoided reading the literature on the subject so that I might make this note just as it presented itself to me—pericardial fluid is not a recognised concomitant of death from asphyxia.

Just after reading the statement above, I had occasion to make a post-mortem on a child of four months where everything pointed to overlying. The right heart was full and the lungs were engorged. There was an ounce of fluid in the pericardium. No signs of inflammation could be discovered. However, the evidence of overlying was so strong that I felt compelled to look upon the presence of fluid as secondary. A few weeks afterwards I made a post-mortem on a baby of six weeks who was found dead in bed with the parents in the early morning. In this case the pericardium was half full of fluid and there was also a small quantity in the left pleural sac. Here I thought death was due primarily to the presence of pleural and pericardial fluid.

Following on these comes a case which makes it appear to me that pericardial fluid may be a result of death from asphyxia.

A girl of sixteen years fell off a 'bus, hitting her head badly. She got up and with some slight assistance walked home. Next morning she attended a dispensary as out-patient and it was thought that she had escaped serious injury. However, in the evening I was called in just in time to witness her death. This was immediately due to respiratory failure. The heart continued to beat for some minutes after the cessation of respiration.

The post-mortem examination revealed a fracture in the right temporal region, with a large extra-dural clot, which had exerted considerable pressure in the region of the Sylvian fissure. The motor area had escaped direct pressure. The right heart was full and there were infarctions at the apices of both lungs. This and what I observed at the time of death points indubitably to asphyxia as the immediate cause of death. She was in good health previous to the accident and was apparently well enough for some time afterwards.

In the pericardial sac there was an ounce and a half of fluid. No inflammatory signs were observed. My conclusion is that pericardial fluid may be present as a result of asphyxia, however produced. The origin of the fluid is probably, at least to some extent, mechanical. Anyway, that is a view which commends itself when one thinks of a strong and vigorous heart struggling desperately to overcome an insuperable difficulty.

